# Exploring the adoption of Schwartz Center Rounds as an organisational innovation to improve staff well-being in England, 2009–2015

**DOI:** 10.1136/bmjopen-2016-014326

**Published:** 2017-01-05

**Authors:** Glenn Robert, Julia Philippou, Mary Leamy, Ellie Reynolds, Shilpa Ross, Laura Bennett, Cath Taylor, Caroline Shuldham, Jill Maben

**Affiliations:** 1 The Florence Nightingale Faculty of Nursing and Midwifery, King's College London, London, UK; 2 The King's Fund, London, UK; 3 Independent Consultant, London, UK

**Keywords:** QUALITATIVE RESEARCH, EDUCATION & TRAINING (see Medical Education & Training), Schwartz Rounds

## Abstract

**Objectives:**

Schwartz Center Rounds (‘Rounds’) are a multidisciplinary forum in which healthcare staff within an organisation discuss the psychological, emotional and social challenges associated with their work in a confidential and safe environment. Implemented in over 375 North American organisations, since 2009, they have been increasingly adopted in England. This study aimed to establish how many and what types of organisations have adopted Rounds in England, and to explore why they did so.

**Setting:**

Public healthcare organisations in England.

**Participants:**

Secondary data analysis was used to map and profile all 116 public healthcare organisations that had adopted Rounds in England by July 2015. Semistructured telephone interviews were conducted with 45 Round coordinators within adopting organisations.

**Results:**

The rate of adoption increased after a major national report in 2013. Rounds were typically adopted in order to improve staff well-being. Adopting organisations scored better on staff engagement than non-adopters; among adopting organisations, those performing better on patient experience were more likely to adopt earlier. Most adoption decision-making processes were straightforward. A confluence of factors—a generally favourable set of innovation attributes (including low cost), advocacy from opinion leaders in different professional networks, active dissemination by change agents and a felt need to be seen to be addressing staff well-being—initially led to Rounds being seen as ‘an idea whose time had come’. More recent adoption patterns have been shaped by the timing of charitable and other agency funding in specific geographical areas and sectors, as well as several forms of ‘mimetic pressure’.

**Conclusions:**

The innate attributes of Rounds, favourable circumstances and the cumulative impact of a sequence of distinct informal and formal social processes have shaped the pattern of their adoption in England.

**Strengths and limitations of this study**

- Theoretically informed study of pattern of adoption of an organisational innovation over time using a complete national sample data set.
- Explores associations between timing of adoption and three measures of organisational performance.
- The ‘date of adoption’ used in the analysis can only be an approximation, given that an adoption ‘decision’ typically comprises a combination of decision points.
- If—over time—all NHS hospitals and hospices in England adopt Rounds, a greater number of the adopters described here would be categorised as ‘innovators’ or ‘early adopters’.
- We did not survey or interview non-adopting organisations.

## Background

Few organisational interventions exist to support staff with the emotional aspects of providing patient care and to sustain compassion in practice, and even fewer have been evaluated. The Schwartz Center for Compassionate Healthcare was founded in 1995 in memory of the late Kenneth Schwartz, an American attorney who had been diagnosed with lung cancer and who, during his treatment, observed how important the connection was between caregivers and patients. The aim of the Schwartz Center is to promote compassionate care (defined as ‘patients and their caregivers relating to one another in a way that provides hope to the patient, support to caregivers and sustenance to the healing process’); it developed a practical tool in the form of Schwartz Center Rounds (henceforth referred to as ‘Rounds’) with the original aim of strengthening the caregiver–patient relationship.

Rounds are an organisation-wide, multidisciplinary forum in which clinical and non-clinical staff from across the healthcare setting (eg, a hospital or hospice) come together to discuss the human dimensions of caring for patients. Rounds are different from traditional medical rounds in that they are not focused on a patient's treatment; rather staff are encouraged to openly discuss the psychological, emotional and social challenges associated with their work in a confidential and safe environment. Advocates of Rounds argue that caregivers are better able to make personal connections with patients and colleagues when they have greater insight into their own responses and feelings, and have an opportunity and space to process these feelings by listening and sharing their experiences with colleagues. Each round has a topic or patient focus and a title that is shared in advance in publicity material.

Each Round lasts for 1 hour, is preceded by food and begins with a multidisciplinary panel presentation. Each panel member focuses on their own experiences in relation to the emotional and psychological impact of caring for patients and their families and any issues arising in terms of working with colleagues. Together with a clinical lead, a trained facilitator guides the discussion of emerging themes and issues, allowing time and space for the audience and panel to reflect and talk about similar experiences that they have had. Attendance is voluntary, open to all and staff attend as many or as few Rounds as they would like, with attendance varying from 10 to over 100. In 2015, Rounds were running in ∼375 healthcare organisations in the USA and 1 in Canada.

In 2009, the (now) Point of Care Foundation (POCF) in London contracted with the Schwartz Center Boston to work with two English acute hospitals to pilot Rounds; their first known expansion beyond North America. Initially, there was a concern that Rounds might not be culturally appropriate, hence the small-scale pilots in 2009 which explored whether clinicians in England would be willing to reflect on and share their personal experiences as openly as their counterparts in the USA.^1^ The Foundation's guidance suggested that Rounds should ideally be run monthly, stipulating a minimum of nine Rounds are held each year. Under the terms of the contract, hospitals and hospices have to meet certain criteria.^2^

The clinical lead and facilitator, supported by steering group members, are responsible for identifying individuals to present topics and cases, manage the advance publicity for the Rounds and evaluate them on an ongoing basis (by analysing feedback sheets given to attendees). The contract for training and support to run Rounds for 2 years currently costs £15 960 (for ‘large’ organisations, defined as having more than 1000 staff or operating several sites over a wider area) and £4500 for small organisations; after this period, organisations can move to ‘membership level’ (a further £3780 for 2 years for ‘large’ organisations and £1680 for small organisations), providing access to resources, an annual conference, webinars and links to the ‘Schwartz Community’. Once the Rounds are established, it is anticipated that they will run indefinitely, embedded into organisational routines.

Following serious failings in care at an acute hospital in England, Rounds were identified as a potential innovation to help bring staff together in the high-profile report into ‘The Mid Staffordshire NHS Foundation Trust Public Inquiry’ (the ‘Francis report’) in February 2013. The report stated that: “A sense of there being one team for the patient should be fostered where possible. One way to help in this might be to involve staff of all backgrounds in case reviews, clinical audit, and in overall team meetings … One method whereby this has been achieved has been by Schwartz rounds (sic).”^3^ As part of the UK government's response to that report in May 2013, £600 000 funding was granted to the POCF over 2 years in order to promote and spread Rounds across the NHS through training and mentorship.^4^ However, it is important to note that Rounds are not prescribed or in any way mandatory in England.

To date, little is known about the organisations that provide Rounds in the UK or how or why they made the decision to adopt this particular organisational innovation. Here we: first, describe how many and what types of organisations were running Rounds in England (as at July 2015); second, report when these organisations adopted Rounds; and, third, compare the performance of adopting and non-adopting organisations on three selected measures. Finally, we explore how and why organisations had adopted Rounds by September 2014.

## Methods

We used a mixed-methods approach. Our study sample of adopting organisations by mid-July 2015 (n=116) was based on a database of organisations with a contract with the POCF; the sample reported here therefore represents all adopters to that point in time as without a contract organisations could not run Rounds in England. Our sample included 113 public healthcare organisations and hospices as well as 1 medical school, a private hospital and a prison (the latter included in a mental health organisation contract). Time of adoption was defined as the date on which an organisation—known to be running Rounds—was sent a contract as this data set was the most complete. Secondary data were used to profile all healthcare organisations running Rounds by July 2015. We used descriptive statistics to explore organisational and geographical characteristics from this sample (including types of service providers). We examined when these organisations adopted rounds, categorising each in terms of time of adoption by mapping them to the five categories of adopters described by Rogers.^5^Adopters and non-adopters were compared using publicly available information relating to public healthcare organisations as these represented the majority (n=88, 74%) in our sample. Other comparisons for hospices and private hospitals were not possible due to a lack of publicly accessible data. The total of non-adopter public healthcare organisations in England was calculated from the most comprehensive and latest source available: a national staff survey conducted in 2015. From this, we identified 153 organisations in England as non-adopters of Rounds.

We used inferential statistics to profile and compare (1) adopters and non-adopters and (2) organisations within the different adopter categories. This involved identifying possible relationships between the categories (including non-adoption) and organisational performance on three measures:

- rankings from a national accreditation body which range from band 1 (organisations that are the highest priority for inspection) and band 6 (lowest priority);^6^
- a ‘staff overall engagement score’ from a national staff experience survey:^7^ this overall score combines scores of three dimensions of engagement (staff advocacy, motivation and involvement) converting them into an overall index of staff engagement. This score was selected as the overall engagement score has the strongest relationship with the general health and well-being of NHS staff;^8^
- an ‘overall patient experience score’ from a national inpatient survey: this score is derived by calculating the average of five domain scores.^9^

For (B) and (C), we collated adopting organisation results for the specific year of adoption and for each of the 3 years prior to adoption (where available). The χ^2^ test was used to examine relationships between categorical variables. When variables included ordinal data, the Spearman's rho (rs) test was used to identify any correlations. The Mann-Whitney test (U) was used to identify differences between adopters and non-adopters. The significance level for the study was set as p<0.05.

Such mapping and profiling of adopting organisations generates a basic description of the general rate of diffusion of an innovation over time but cannot help explore reasons for—or processes of—adoption.^10^ We therefore also conducted 45 semistructured, audio-recorded telephone interviews with clinical leads or facilitators responsible for leading Rounds as at September 2014. Twenty-eight of the interviewees were from acute hospitals, 10 from hospices and 7 from mental health/community organisations. Interviews were conducted between February and August 2015. The interviews explored, for example, when and why interviewees took up their role of Round lead/facilitator, the main reasons for deciding to run Rounds in their organisation and who initiated the introduction of Rounds into the organisation. One further interview was conducted with the chief executive of the POCF in order to explore in more detail the story of how Rounds were brought to the UK. A framework approach was used to organise the interview data. Themes were extracted deductively based on the Diffusion of Innovations model^10^ which was developed through an extensive literature review which led to an evidence-based model for considering the diffusion of innovations in health service organisations (and itself informed by ‘diffusion of innovation’ theory which describes how innovations are communicated and taken up across social systems^5^).

## Results

### How many and what types of organisations are running Rounds?

One hundred and sixteen organisations in England had adopted Rounds by 15 July 2015 (table 1). The majority of these organisations were acute, community and mental health services (n=87, 73%); there were 25 hospices (24%) and 4 (3%) other organisations (an ambulance service, a prison, a university medical school and a private hospital). Over half (n=71, 60%) of the adopting organisations were based in the south with 32 in London (26%). Table 1 shows that 44% of acute organisations had adopted rounds, followed by 28% of Mental Health and Learning Disability organisations and a smaller percentage (18%) of community only services. Approximately 14% of organisations offering hospice care had adopted Rounds.

**Table 1:** Number of adopters and non-adopters in England (July 2015)

Providers	Adopters (no.)	Non-adopters	No. of organisations	% Adoption
NHS England				
Acute (including acute/community combined and Specialist trust)	68	87	155	44%
Mental health/learning disability	16	42	58	28%
Community	3	14	17	18%
Ambulance trusts	1	10	11	9%
Subtotal	88	153	241	36.5%
Hospice	25	172	197	13%
Other*	3	n/a	n/a	n/a
Total	116	325	438	26%

*Prison, university and private hospital.

### When did organisations adopt Rounds?

**Figure 1:**
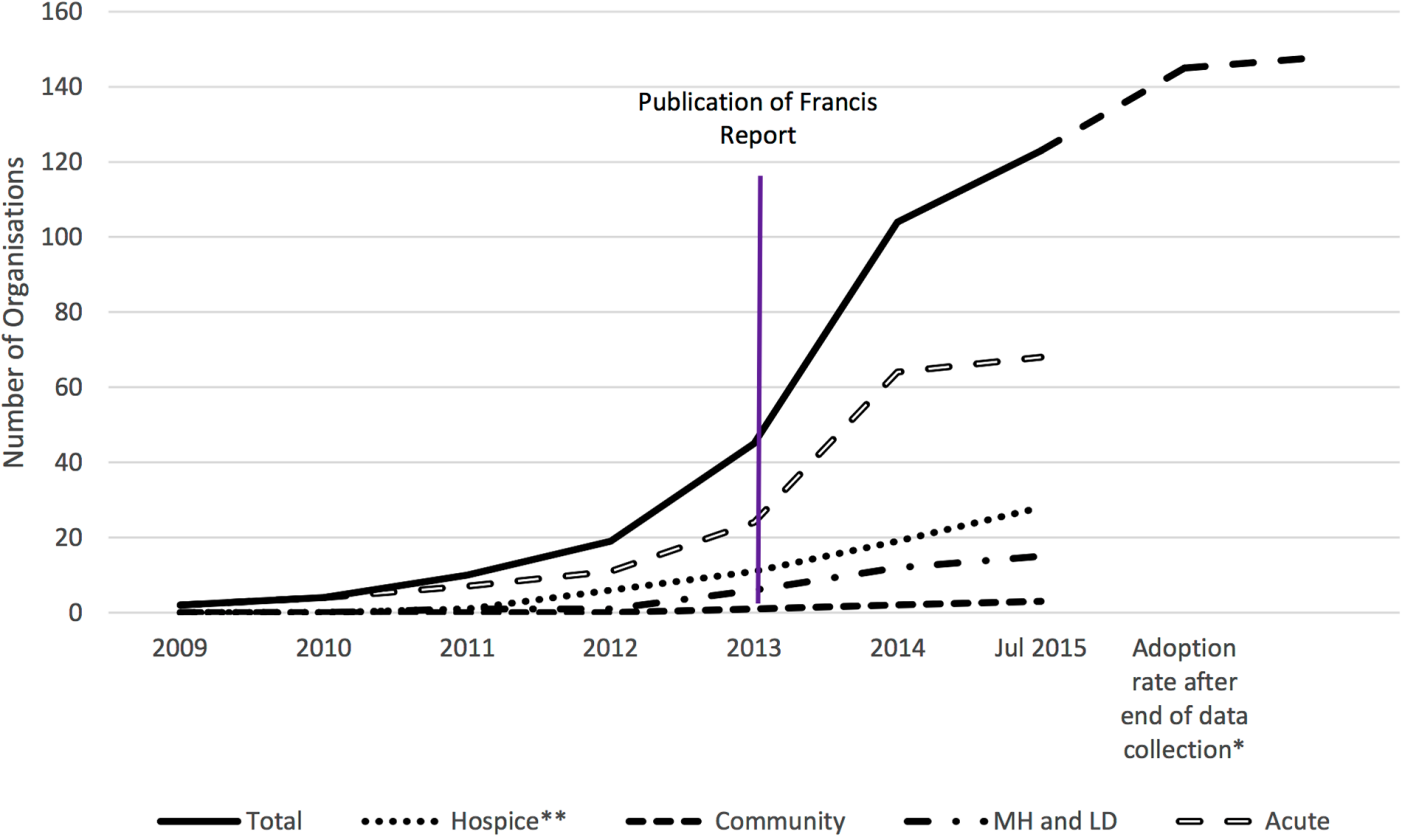
Adoption over time by provider type in England (acute, mental health and learning disability (MH/LD), community*,* hospice). *Dotted curve indicates adoption rate after the end of study's data collection point (mid-July 2015). Data provided by Point of Care Foundation on 9th February 2016. **Hospice UK wide sample.

Figure 1 illustrates the rate of adoption of Rounds by provider type (acute care, mental health and learning disability, community care and hospice). There was a sharp increase in Rounds adoption in latter 2013 and throughout 2014. One possible explanation is the publication of the high-profile Francis Report in February 2013.^3^

Between 2009 and 2012 (prior to the Francis Report), 20 organisations had adopted Rounds. January 2015 saw the highest number of organisations adopting Rounds (n=11) in any month.

### What are the characteristics of organisations adopting Rounds at different times?

Table 2 shows how Rounds adopters were categorised by date of adoption and then mapped onto the categories of adopters outlined by Rogers.^5^

**Table 2:** Adopting organisation grouped by ‘diffusion of innovation’ categories

Diffusion of innovation adopter category	% of adopters	Number of Rounds adopters in this category
Innovators	First 2.5%	3
Early adopters	Next 13.5%	16
Early majority	Next 34%	41
Late majority	Next 34%	41
Laggards	Final 16%	18
Missing data		4
Total adopters*	100%	123

*England, Scotland and Wales.

Using this categorisation, we explored possible relationships between the timing of adoption of Rounds for NHS organisations and the three performance measures described earlier (table 3).

**Table 3:** Timing of adoption and performance measures (for NHS England organisations only)

CQC ratings 2013†	Adopters	Non-adopters	Total	χ^2^
Band 1 (highest risk)	9 (14%)	13 (14%)	22 (14%)	p=0.23
Band 2	12 (19%)	8 (9%)	20 (13%)	
Band 3	8 (13%)	20 (22%)	28 (18%)	
Band 4	8 (13%)	16 (17%)	24 (15.5%)	
Band 5	8 (13%)	16 (17%)	24 (15.5%)	
Band 6 (lowest risk)	18 (28%)	19 (21%)	37 (24%)	
Total	63 (100%)	92 (100%)	155 (100%)	
Staff overall engagement score max score 5	Adopters	Non-adopters	National mean	U
2015	M=3.79 SD±0.11 (n=82, 3.47–4.04)	M=3.77 SD±0.15 (n=159, 3.11–4.03)	3.78	p=0.98
2014	M=3.72 SD±0.12 (n=82, 3.43–4.03)	M=3.70 SD±0.18 (n=159, 2.77–4.02)	3.75	p=0.93
2013	M=3.74 SD±0.13 (n=82, 3.38–4.09)	M=3.69 SD±0.16 (n=159, 2.92–4.06)	3.75	p=0.04**
Inpatient survey overall experience score Max Score 100	Adopters	Non-adopters	National mean	U
2015	M=76 SD±3.7 (n=76, 67–87)	M=76 SD±3.1 (n=129, 71–87)	77	p=0.64
2014	M=75 SD±4.2 (n=76, 65–87)	M=75 SD±4 (n=128, 68–87)	76	p=0.96
2013	M=76 SD±3.8 (n=76, 68–88)	M=76 SD±2.8 (n=129, 70–87)	75	p=0.98

**p<0.05.

†Data available for only 155 NHS England organisations across acute, community and mental health.

M, mean (n, number, Range).

Comparison of adopters and non-adopters did not identify any significant differences apart from staff engagement scores in 2013 when adopter organisations scored higher (M=3.74, SD±0.13) than non-adopters (M=3.69 SD±0.16) (U=5464.5, p=0.04, r=−0.13).

When examining trends within adopters' category over the 3 years before adoption, we identified two weak positive correlations; these indicated that the earlier the organisations adopted rounds, the more likely they were to have better overall patient experience results in 2013 (r_s_=0.25, p=0.02) and 2015 (r_s_=0.24, p=0.03). Overall adopting organisations and organisations adopting rounds earlier (eg, innovators and early adopters) tended to perform better on staff and patient experiences compared with non-adopting organisations or organisations adopting later (late majority, laggards).

### Why were Rounds adopted?

We consistently found that explanations for adoption related to staff well-being (rather than explicitly referring to improving compassionate care for patients):

You need to care for the carers if they're going to care for people. And it's been one of the things I've kind of got rather frustrated in the whole way that the health service works, that it's not very good at caring for the carers. (Clinical lead; cardiologist, acute hospital adopted 2011)

My main reason was because I was wanting to provide more psychological support, emotional support, for staff but I know that from an organisational point of view some of the motivation was around improving working relationships between different teams. (Facilitator; psychologist, acute hospital adopted 2014)

Interviewees were often those championing Rounds adoption; the most vivid passages in our interview data were those describing their own personal experience of Rounds where the emotional charge and effect on participants was very clear. More questioning accounts stood out as exceptions.

We now turn to key components of a conceptual model^10^—specifically developed to explain the adoption of innovations in healthcare service delivery and organisation—in order to explore the story of Rounds in England.

In terms of the attributes of the ‘innovation’ itself, Rogers^5^ originally argued that an individual's perceptions of five attributes of an innovation could predict its rate of adoption; he later highlighted the importance of also considering a sixth attribute (reinvention). Table 4 summarises interviewees' perceptions of Rounds according to these attributes, illustrating each with participant quotations.

**Table 4:** Attributes of Rounds

Attribute (Rogers^5^)	Rounds as an organisational innovation	Example quotations
Relative advantage: degree to which perceived as better than idea it supersedes	Has relative advantage: few alternative organisational interventions and has provenance/branding. Relatively low cost. Many adopting organisations received funding from charities to pay for licence, thereby making Rounds a ‘free good’	“seemed quite a valuable thing, something very different, an opportunity for lots more staff engagement and we all saw it as a very positive thing” (Clinical lead; doctor, acute hospital adopted 2011)“there was recognition that there was something missing or something that could be added around staff engagement” (Clinical lead; doctor, acute hospital adopted 2014)
Compatibility: degree to which perceived as consistent with existing practices and values, past experiences and needs of potential adopters	Highly compatible. Format (large meeting, like medical ‘grand round’) is familiar, if not the content. Staff reflection familiar in clinical supervision, debriefing after incidents. Meeting recognised need as a demonstrable response to Francis Report	“certainly it fitted in with some of the other things I'd done … where we had sessions with clinical teams … it was a way of bringing that clinical supervision or that listening to a bigger audience” (Clinical lead; doctor, acute hospital adopted 2011)“there were links with the Francis report and the findings of that and things that we committed to as an organisation within that” (Facilitator; mental health hospital adopted 2014)
Complexity: the degree to which perceived as difficult to understand and use	Perceived as complex but having off-the-shelf model and package offered by POCF has helped. Nonetheless are difficult to describe without experiencing/observing them. Do not require service restructuring or large-scale staff training (only for facilitators); can be perceived as a ‘bolt-on’ to existing practices	“there is initially getting your head round what [Rounds] were and what kind of difference they would make … They are hard to describe. You need to go witness” (Facilitator; psychologist, acute hospital adopted 2009)“If they haven't seen it before, it's quite difficult to describe, it really sounds too simple … they couldn't really picture how it's going to work” (Facilitator; doctor, acute hospital adopted 2013)
Trialability: the degree to which an innovation may be experimented with on a limited basis	Not trialable due to licensing arrangements, although piloting undertaken in some organisations and can amend incrementally on monthly basis (eg, time of day, location). Can be stopped relatively easily	“We started off as a small pilot in the south of the county when we were looking at how to bring rounds in we thought ‘OK, we'll start with a small geographical area” and we set them up for different pilots, we ran one here, which is an Acute in-patient unit … so that over the 6 months we could start off on a small scale, learn from our mistakes etc before we rolled them out to the wider Trust” (Clinical lead; facilitator, community hospital, adopted 2011)“in a small organisation like ours why do we need something like that? That was the big thing right from the start … we were nervous about it and that was the biggest challenge in setting it up” (Deputy clinical lead and facilitator; hospice adopted 2013)
Observability: the degree to which the results of an innovation are visible to others	Observable in that can attend and see Rounds as introduced ‘successfully’ elsewhere. However, results/impact difficult to evaluate. Can be deferred either on a ‘we will know when we see’ it basis and/or use of simple metrics (eg, numbers attending and diversity/representativeness of groups of staff)	“seen it as a very powerful thing in America and felt it would be a good, positive thing for us to do here” (Clinical lead, hospice adopted 2013)“it's very hard to give you tangible … hard quantitative type of evidence, but what is clear is that when staff attend, the evaluations after the event are incredibly positive” (Director of Nursing; acute hospital, adopted 2013)“it was so powerful … I thought it would be great from the effect of having seen a live Round” (Clinical lead, acute hospital adopted 2015)“lots of people wanted to know about what the outcomes would be and how are we going to measure it … there was a lot of anxiety” (Facilitator; palliative care, acute hospital adopted 2013)
Reinvention: the extent to which the innovation can be changed or modified by the user in the process of adoption and implementation	In formal licensing terms not very modifiable but in practice, more so. There is a set format to Rounds but is variation in relation to many aspects of structure and function that are permitted within the terms of licence	“So we were a little bit worried at the beginning when it was quite prescriptive … I don't know whether we're meeting our contract or not but hopefully we're doing it so it's sustainable in the future” (Clinical lead and facilitator; manager/physiotherapist, community hospital adopted 2014)“looking at it we can do them in a more lean way but still retain the essence of Schwartz” (Clinical lead; psychiatrist, community hospital, adopted 2014)

With the exceptions of ‘complexity’ and ‘trialability’ (and possibly aspects of ‘observability’), consideration of Rogers attributes with regard to Rounds suggests that they would be an organisational innovation that is likely to be adopted. With regard to reinvention, it is noteworthy that the most common reason for introducing Rounds was attending to staff well-being through a form of emotional support. This differs from the original aims of Rounds—as developed in the USA—where they were commonly and explicitly framed as an innovation for improving compassionate care for patients.

In terms of the role of specific individuals, interviewees in our ‘innovator’ organisations were a psychologist and a medical director who had visited the Schwartz Centre in the USA (with the POCF) and who became strong advocates for Rounds. Later, a majority of interviewees reported it was a consultant (in various specialisms) who had initiated the introduction of Rounds followed equally by Medical Directors or psychologists. Senior nurses (up to Director of Nursing level) and other individuals (patient experience leads and social workers) were also identified as important in initiating the adoption of Rounds.

Interviewees described personally gathering support for Rounds among a few colleagues before presenting the idea to their board or senior management team. A formal proposal or business case was not always made (in a minority of cases, this had consequences; eg, a lack of administrative support or ongoing funding), although the contract with the POCF stipulates that adopting organisations provide a letter of support from their chief executive. In those cases where a Board-level decision was made, the adoption decision was often little more than a formality; there were few cases where Rounds were rigorously ‘assessed’ as part of a decision-making process. If adoption was proposed by a group of enthusiasts who were willing to manage implementation and funding could be secured for most of the set up costs (as in the case of hospices where two national charities made such funding available, further emphasising the ‘relative advantage’ of Rounds in this sector), then Rounds were perceived as ‘worth trying’. Overall, it appeared to be relatively straightforward to gain approval for—and there was little resistance to—introducing Rounds in the majority of organisations.

It was also striking how many interviewees spoke positively of having visited a ‘neighbouring hospital’ that was already running Rounds prior to an adoption decision being made; this was a mandatory step in the licensing process. That staff were required to visit others and observe a Round in practice perhaps further reflects the perceived ‘complexity’ of the innovation (table 4). Such connections were facilitated—not through formal organisational ties—but rather via horizontal professional (eg, clinical or psychology) networks or mediated by the POCF. Later, change agents had a greater coordinating role in the formal dissemination of Rounds, particularly post-Francis Report publication and the Government's response. The POCF undoubtedly played a central role; its chief executive spoke on the topic of organisational culture during the public enquiry at a seminar chaired by Sir Robert Francis himself at which she mentioned the value of Rounds. The chief executive and colleagues also spoke about Rounds at many conferences post-Francis and she was invited to apply for a grant by the government for funds to support their wider dissemination.

Illustrations of the typical ‘readiness for change’ within organisations included a small group of individuals—who wanting to implement a form of support for staff that specifically addressed the emotional impact of their work—discovered that this aligned very well (ie, was compatible) with emerging organisational priorities:

The Trust I know was reflecting on itself as an organisation and … had got strategies to be a compassionate employer … and it [Rounds] sat very, very well with some of the ambitions that the Trust was developing at the time and how it went about its business really… (Clinical lead; doctor, acute hospital adopted 2014)

We took it to the board at a time when the need to be mindful of the emotional impact work could have on employees was already part of the conversation … (Clinical lead; nurse, community hospital adopted 2011)

However, interviewees also suggested that organisational readiness was sometimes a result of rather different motivations (and on occasion, these were shaped by external pressures):

A surprising amount of documentation about us as an organisation includes Schwartz Rounds … in an ill-formulated way, it sort of ticks boxes of, are we doing the right thing by staff? Schwartz Round: tick. (Clinical lead; elderly care, acute hospital)

It was something to do with part of the conditions of coming out of special measures … and we received some additional funding in order to do that. (Clinical lead and facilitator; medical, acute hospital adopted 2014)

Finally, with reference to the outer context, there was little mention of any factors beyond the Francis Report, the dissemination activities of the POCF and (for later adopters) the availability of funding from recognised national charities. The publication of the Francis Report was mentioned—often in vague terms—as significant (“*I think it was, if I remember, to do with the Francis Report and it had been mentioned in the Francis Report, I think that's right…*”), but as the Director of the POCF commented: “*he [Sir Robert Francis] didn't endorse them [Rounds]; he just said, ‘here they are, something worth thinking about*.’ Nonetheless, there were unequivocal external expectations that organisations would act (somehow) in response to the Report and references to presentations or conferences where staff from the POCF proposed Rounds as an intervention in this context were commonly mentioned as important events.”

Overall, there was a confluence of factors—a generally favourable set of innovation attributes (including low cost), advocacy from opinion leaders in several professional networks, active dissemination by credible change agents, informal visits to other organisations and the felt need to be seen to respond to the Francis Report (particularly its emphasis on improving staff well-being)—allowed Rounds to be seen as an idea whose time has come; “*it just felt like the right thing to do,*” “*a no-brainer*” as two of our interviewees commented.

## Discussion

Our secondary analyses of selected performance metrics found a small number of weak associations between these and the timing of adoption at the organisational level. However, we found that the pattern of adoption of Rounds to date has not been driven in a significant way by any ‘felt need’ to respond to ‘poor’ performance. There was little evidence that those advocating for Rounds were part of formal, strategic search attempts to address such issues.

Nor has the adoption of Rounds been driven by a strong evidence base (this evidence deficit is common to many staff well-being interventions). Anecdotally—including in our interviews—there is considerable advocacy based on proponent's personal experiences of participating in Rounds. However, there was only one US and one UK evaluation published by July 2015. The former comprised retrospective surveys of attendees at 6 sites offering Rounds for more than 3 years and prospective surveys of attendees at 10 new Rounds sites that had held more than 7 Rounds, while the latter focused on the feasibility of transferring Rounds to England in 2 pilot organisations; both suggested potential benefits.^1^ ^11^ One further (positive) case study in a US hospital has subsequently been published.^12^ Given that several staff well-being initiatives are likely to be underway in a single healthcare organisation, attributing any improvement in staff well-being to a single intervention is likely to be problematic. Rounds may (perhaps understandably) be viewed as a ‘common sense’ intervention, but this does not necessarily undermine the need for rigorous evaluation that also considers wider systemic effects and potential unintended consequences.^13–15^ For example, we do not know what proportion of staff—or which staff—may need to attend Rounds (and over what period) in order to maximise the impact of this organisational innovation.

There was a marked increase in the numbers of organisations adopting Rounds in 2013 and 2014 lending support to the hypothesis that organisations were responding to the publication of the Francis Report. However, the sole, very brief reference to Rounds is rather oblique; it appears on page 1394 of volume 3 under the topic of ‘teamwork and leadership’.^3^ No mention is made of Rounds in the 125-page executive summary of the Report nor in any of the Inquiry's 290 recommendations. In the British government's 385-page response to the Francis Report in January 2014, there are two—again brief—references to Rounds; the first suggesting they ‘can help staff come to terms with the realities of caring’ and the second describing the funding grant to the POCF.^4^ This suggests less of a role for the publication of the Report and the government's response than might be assumed.

The adoption of Rounds—as with other staff well-being interventions—has not been driven by government mandate or evidence-based decisions, but it does appear that the nature of the social processes influencing adoption of Rounds in England has changed over time. From 2009 until 2013 (and the publication of the Francis Report), adoption was largely driven by ‘passive spread’;^10^ what Rogers originally defined as diffusion, namely that an innovation is communicated informally over time among individual members of a social system.^5^ In the case of Rounds, this highly social process^16^ was mediated through professional networks—sometimes supplemented by mentions of Rounds in articles and policy commentaries—and led to 20 organisations (the majority in London and the south of England) adopting Rounds. As Harris *et al*^17^ have argued, more research is needed to understand how the source of an innovation shapes potential adopters' responses, but in this case, we found no evidence of ‘importing’ the idea from the USA to be a barrier to adoption. Of potential significance is that, rather than Rounds being ‘reinvented’ by healthcare organisations in England, the reframing of their purpose—less explicitly relating to teaching compassionate care and with a greater focus on staff well-being—was led by influential individuals who were key in transferring (and translating) this US innovation; they were adapted during the training of facilitators in ‘early adopter’ organisation (before they were widely adopted).^18^ Then, in the aftermath of the Francis Report, the efforts of the POCF as change agents advocating for Rounds as an organisational response led eventually to a more active process with planned, targeted dissemination efforts successfully persuading larger numbers of organisations to adopt. Finally, the adoption of Rounds is now perhaps entering a third phase where some organisations—particularly acute hospitals—are adopting Rounds for additional reasons; as they became perceived as something that a hospital ought to be ‘seen’ to be doing. In these cases, Rounds have taken on symbolic value as a means of identity management and meeting external expectations (as illustrated above: ‘ticks boxes’ and ‘part of the conditions’).

DiMaggio and Powell^19^ describe this third phase as institutional isomorphism: a ‘constraining process that forces one unit in a population to resemble other units that face the same set of environmental conditions’. They identified three mechanisms underlying such a process each of which are now playing a part in the ongoing story of Rounds in England. First, mimetic processes are driven by uncertainty which encourages imitation; uncertainty in the case as to how to respond to the need to improve staff well-being is leading organisations to visit and copy more innovative peers. Second, normative pressures are brought about by professions; as noted, interorganisational professional networks were highly relevant in the case of Rounds (as they have been in the adoption of other interventions such as the Productive Ward programme^20^). Of less importance generally—but pertinent in a minority of later adopting organisations—was the role of ‘coercive isomorphism’: pressures from other organisations on which they are dependent (in this case from external regulators—and pressures real or imagined—as evidenced in the example above). As Dixon-Woods *et al*^14^ suggest, some of the later adopters in our study (performing less well relative to others as noted) may have turned to Rounds as ‘a defence against anxiety, to guard against criticism that any failing was due to non-adoption’. Or, as DiMaggio and Powell^19^ argue: “Organisations are increasingly homogeneous within given domains and increasingly organized around rituals of conformity to wider institutions.” Organisations in England now perhaps find themselves as having to have a good reason for not adopting Rounds.

There are four limitations to our study. First, for pragmatic reasons, we used the date a contract was sent to an organisation as a proxy for their ‘date of adoption’; this can only be an approximation, given that an adoption ‘decision’ typically comprises a combination of decision points.^10^ ^20^ ^21^ Second, it is important to note that if—over time—all NHS hospitals and hospices in England adopted Rounds and Rogers' categories were reapplied, a greater number of the adopters described here would be categorised as ‘innovators’ or ‘early adopters’. Third, we did not survey or interview non-adopting organisations. As noted in relation to other interventions, sometimes non-adoption or resisting adoption can be the right thing to do in certain local contexts,^14^ where, for example, other priorities are deemed more urgent to address. Finally, as others have argued,^21^ ^22^ further insights into adoption and implementation processes could be gained through theoretically informed longitudinal, qualitative studies; in this regard, a national evaluation of Rounds in England is currently ongoing.^15^

## Conclusions

Significant numbers of healthcare organisations in England adopted Rounds during the period 2009 to mid-July 2015, albeit with wide geographical variations that differ by type of organisation and with the rate now seemingly slowing. We found weak associations, first, between adopting organisations having higher levels of staff engagement than non-adopters and, second, earlier adopters scoring higher than later adopters on patient experience measures.

While there was a sharp increase in adoption after a major national report recommended Rounds as an intervention, the use of Rounds in England to date has been shaped by innate attributes of this innovation, favourable circumstances and the cumulative effect of a sequence of social processes. Initially, these comprised informal diffusion among different professional networks and then more formal planned dissemination activities, whereas latterly, ‘mimetic pressures’—including signs of a felt need to conform to the expectations of external organisations—have become influential.
